# Data on necrotic and apoptotic cell death in acute myocardial ischemia/reperfusion injury: the effects of CaMKII and angiotensin AT_1_ receptor inhibition

**DOI:** 10.1016/j.dib.2016.03.017

**Published:** 2016-03-10

**Authors:** Tomas Rajtik, Slavka Carnicka, Adrian Szobi, Zoltan Giricz, Jin O-Uchi, Veronika Hassova, Pavel Svec, Peter Ferdinandy, Tanya Ravingerova, Adriana Adameova

**Affiliations:** a Department of Pharmacology and Toxicology, Faculty of Pharmacy, Comenius University in Bratislava, Slovak Republic; b Institute for Heart Research, Slovak Academy of Sciences & Centre of Excellence, SAS NOREG, Bratislava, Slovak Republic; c Cardiometabolic Research Group, Department of Pharmacology and Pharmacotherapy, Semmelweis University, Budapest, Hungary; d Department of Medicine, Jefferson Medical College, Thomas Jefferson University, Philadelphia, USA; e PharmaHungary Group, Szeged, Hungary

**Keywords:** Myocardial ischemia/reperfusion injury, CaMKII inhibition, AT_1_ receptor blockade, Apoptosis, Necrosis

## Abstract

Content of particular proteins indicating cellular injury due to apoptosis and necrosis has been investigated in ischemic/reperfused (IR) hearts and ischemic/reperfused hearts treated with CaMKII inhibitor and/or AT_1_ receptor inhibitor. This data article provides information in support of the original research article “Oxidative activation of CaMKIIδ in acute myocardial ischemia/reperfusion injury: a role of angiotensin AT_1_ receptor-NOX2 signaling axis” [1].

**Specifications Table**

**Table:** 

Subject area	*Biology*
More specific subject area	*Myocardial ischemia/reperfusion injury; Cellular injury; Protein kinase*
Type of data	*Figures, Text*
How data was acquired	*Immunoblotting, Chemiluminescence*
Data format	*Raw, Analyzed*
Experimental factors	*SDS-PAGE, Western blotting in samples of left ventricles of the hearts subjected to ischemia/reperfusion injury with and without angiotensin AT*_*1*_*receptor inhibitor and CaMKII inhibitor*
Experimental features	*Analysis of certain proteins indicating cellular injury due to apoptosis and necrosis*
Data source location	*Bratislava, Slovak Republic*
Data accessibility	*Data is supplied in this article*

**Value of the data**

• The data can be useful for other researchers investigating signaling pathways associated with CaMKII and AT_1_ receptor activation under setting of ischemia/reperfusion in the heart.
• These data also provide significant contribution in understanding of pharmacological properties of AT_1_ receptor inhibitors, drugs used in patients at high cardiovascular risk.

## 1 Data

**Fig. 1:**
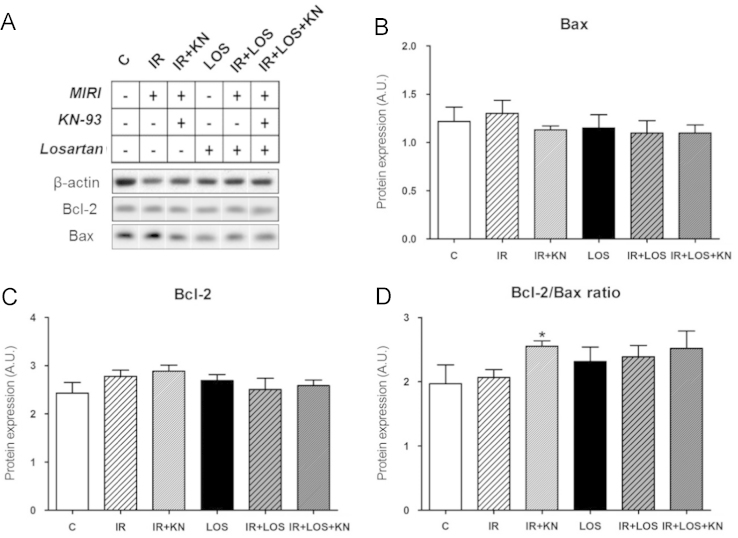
Expression of Bcl-2 and Bax. (a) Representative western blots (b) Bcl-2 and (c) Bax expression (d) Bcl-2/Bax ratio. The values are expressed as the means±S.E.M. (*n*=5–7 hearts per group). ^⁎^*P*<0.05 vs. IR group.

**Fig. 2:**
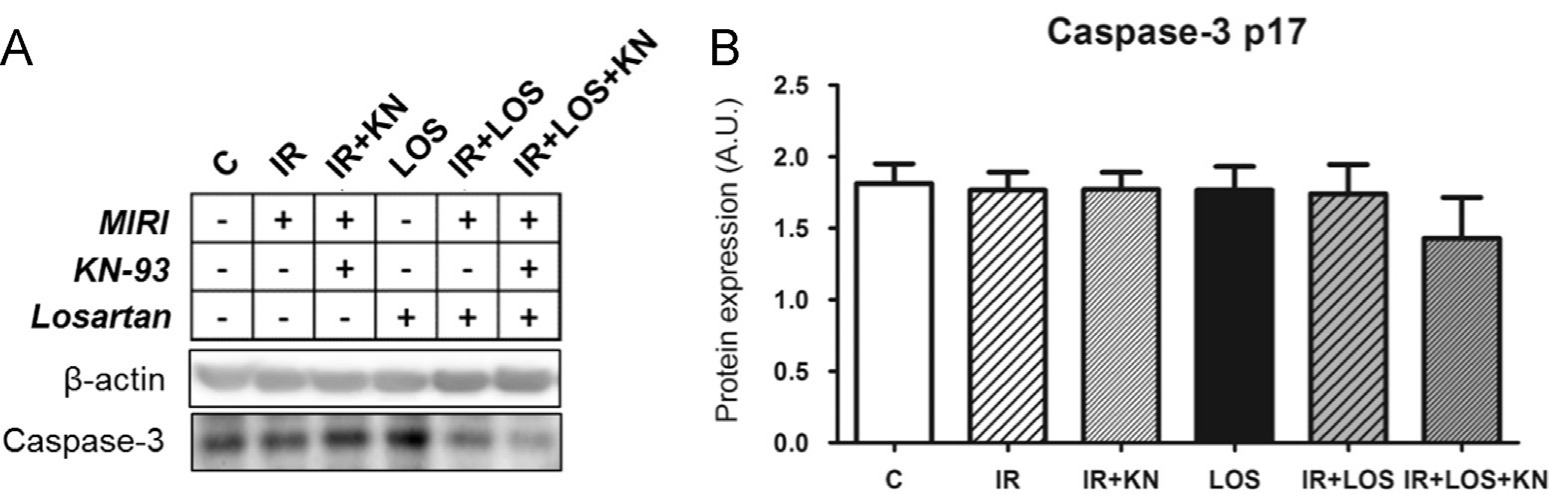
Expression of cleaved caspase-3. (a) Representative blots (b) content of cleaved caspase-3. The values are expressed as the means±S.E.M. (*n*=5–7 hearts per group).

**Fig. 3:**
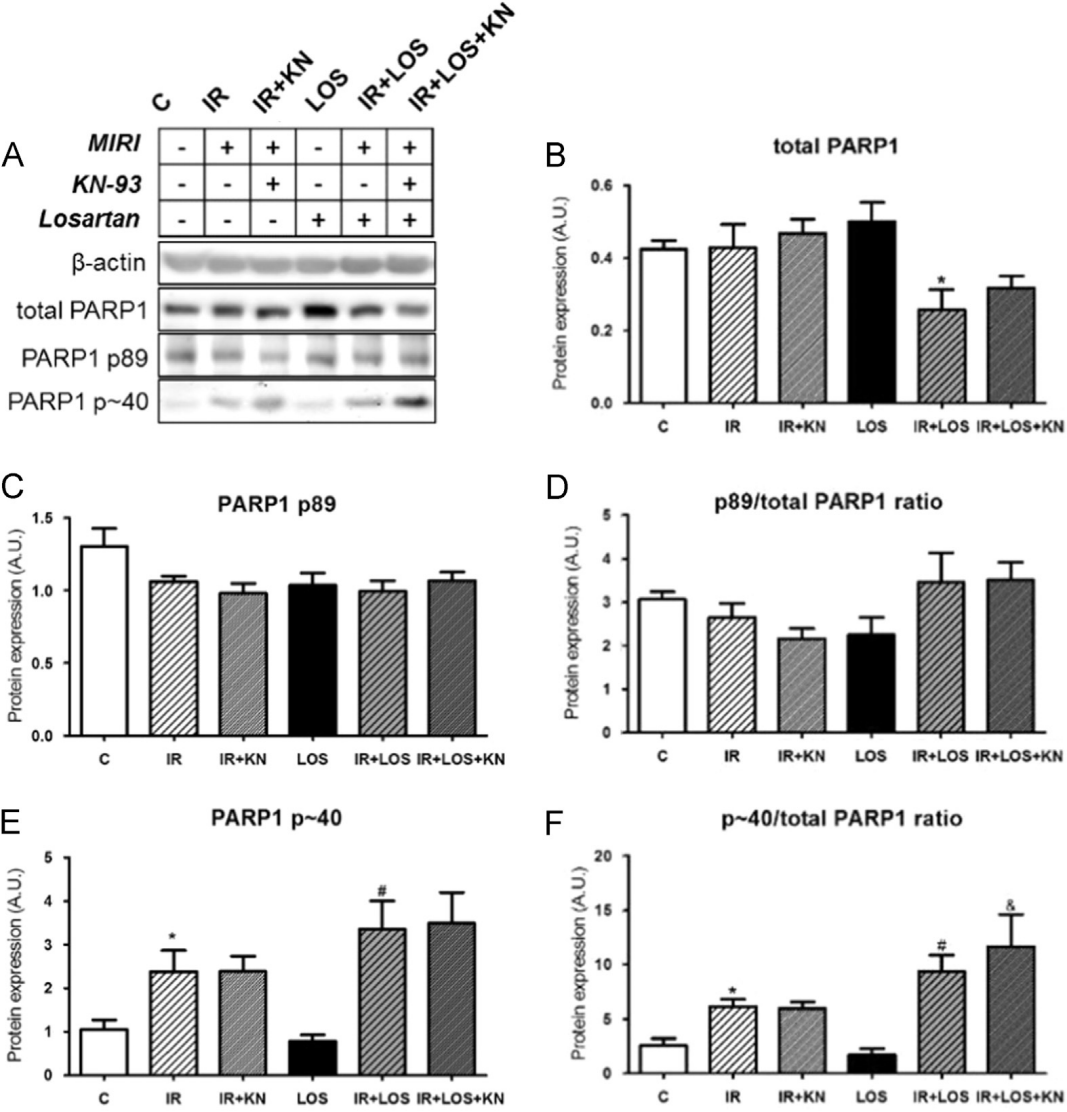
Expression of total and cleavage fragments of PARP1. (a) Representative blots, (b) expression of total protein, (c) content of apoptotic PARP1 p89 fragment, (d) apoptotic p89 fragment to total PARP1 ratio, (e) necrotic PARP1 *p*~40 fragment and (f) ratio of necrotic PARP1 fragment to total PARP1. The values are expressed as the means±S.E.M. (*n*=5–7 hearts per group). ^⁎^*P*<0.05 vs. C; ^#^*P*<0.05 vs. LOS group; ^&^*P*<0.05 vs. IR+KN.

In ischemic/reperfused (IR) group treated with CaMKII inhibitor, but not with AT_1_ receptor inhibitor, Bcl-2/Bax ratio was significantly increased (Fig. 1) and the expression of caspase-3 and 89 kDa apoptotic fragment of PARP1 was changed by neither CaMKII inhibition nor angiotensin AT_1_ receptor inhibition (Fig. 2). ~40 kDa necrotic fragment of PARP1 was upregulated in the IR group and no intervention was able to normalize these levels (Fig. 3). In the double-treated IR hearts, the levels of individual apoptotic and necrotic did not differ from those observed in either of mono-treated IR; however, the *p*~40PARP1/total PARP1 ratio was increased when compared to the IR group treated with CaMKII inhibitor alone.

## 2 Experimental design, materials and methods

Samples from the untreated and losartan and/or KN-93-treated hearts subjected to ischemia/reperfusion injury were used to examine protein expression by immunoblotting.

Proteins were separated using the SDS-PAGE and transferred to PVDF membrane (EMD Millipore, USA). The primary antibodies used were Bcl-2 (Sigma-Aldrich, USA), Bax (Cell Signaling, USA), PARP1 (Cell Signaling, USA), Caspase-3 (csp-3) p17 (Santa Cruz Biotechnology, USA) and β-actin (Sigma-Aldrich, USA). A HRP-conjugated donkey anti-rabbit antibody (GE Healthcare Life Sciences, UK) was used as the secondary antibody. The signals of proteins were detected using enhanced chemiluminiscence (Luminata, EMD Millipore, USA) and the blots were acquired using the MyECL Imager (Thermo Scientific, USA).

Data are expressed as the means±S.E.M. Differences between variables with normal distribution were statistically analyzed by ANOVA with Newman–Keuls post-hoc test. Differences were considered as significant at *P*≤0.05*.*
