# Supplementary material for: Data on necrotic and apoptotic cell death in acute myocardial ischemia/reperfusion injury: the effects of CaMKII and angiotensin AT1 receptor inhibition
Source: Data Brief. 2016 Mar 10;7:730–4. doi: 10.1016/j.dib.2016.03.017 (PMC4802543; doi:10.1016/j.dib.2016.03.017)
Supplement: Supplementary file 1 — Supplementary material [file mmc1.docx]

***Data in Brief***

21.1.2016

Dear Dr. Hao-Ran Wang,

Along with submitting our article “*Data in support of oxidative activation of CaMKIIδ in acute myocardial ischemia/reperfusion injury: a role of angiotensin AT1 receptor-NOX2 signaling axis”*, we would like to declare that there is no conflict of interest.

Yours sincerely,

Dr. Adriana Adameova
